# National program for family planning and primary health care Pakistan: a SWOT analysis

**DOI:** 10.1186/1742-4755-10-60

**Published:** 2013-11-22

**Authors:** Mohammad Salim Wazir, Babar Tasneem Shaikh, Ashfaq Ahmed

**Affiliations:** 1 Department of Community Medicine, Ayub Medical College, Abbottabad, Pakistan; 2 Department of Health Systems & Policy, Health Services Academy, Islamabad, Pakistan

**Keywords:** SWOT, Primary healthcare, Human resources for health, Management information system, Lady health worker, Vertical program, Developing countries, Pakistan

## Abstract

**Background:**

The National Program for Family Planning and Primary Healthcare was launched in 1994. It is one of the largest community based programs in the world, providing primary healthcare services to about 80 million people, most of which is rural poor. The program has been instrumental in improving health related indicators of maternal and child health in the last two decades.

**Methods:**

SWOT analysis was used by making recourse to the structure and dynamics of the program as well as searching the literature.

**SWOT analysis:**

*Strengths* of the program include: comprehensive design of planning, implementation and supervision mechanisms aided by an MIS, selection and recruitments processes and evidence created through improving health impact indicators. *Weaknesses* identified are slow progress, poor integration of the program with health services at local levels including MIS, and de-motivational factors such as job insecurity and non-payment of salaries in time. *Opportunities* include further widening the coverage of services, its potential contribution to health system research, and its use in areas other than health like women empowerment and poverty alleviation. *Threats* the program may face are: political interference, lack of funds, social threats and implications for professional malpractices.

**Conclusion:**

Strengthening of the program will necessitate a strong political commitment, sustained funding and a just remuneration to this bare foot doctor of Pakistan, the Lady Health Worker.

## Background

Role of community health workers in primary health care provision has now been well established and oft-documented for improving the health of the population, particularly in countries which face serious dearth of skilled human resource [1]. Commonly known as *“Lady Health Workers’ Programme”*, National Program for Family Planning and Primary Health Care was launched in April 1994 by the then Prime Minister of Pakistan, Mohtarama Benazir Bhutto, as a major initiative to provide universal health coverage to the people of Pakistan fulfilling the Program of Action brought from ICPD held in Cairo [2]. Her motivation led to one of the largest and successful community based programs in the world providing primary health care services to more than 80 million people of the country at their door steps [3,4]. The main thrust of the program is to extend the outreach services to the communities through selection and training of 100,000 Lady Health Workers (LHWs) from all over the country, in a phase-wise manner. The program was designed as an integral part of existing health care delivery system of the country through locally identified literate female workers who were trained on primary health care and placed in the communities to which they belonged. The LHWs are trained to provide essential maternal and child health care including family planning, management of minor and common ailments and imparting health education [5].

Since the Alma Ata declaration in 1978, global efforts to improve access to primary health care for poor and vulnerable populations had started [6]. Latin America, Tanzania, Mozambique, and China were the leading countries where large scale community based health programs were initiated. The concept of “barefoot doctors” during 1960s in China has a history of providing basic health care to rural populations. Deployment of community health workers (CHWs) has been a recognized strategy to provide basic health care at community level and to bridge the gap between community and the health system in low and middle income countries [3].

During 1970s and 80s, health indicators related to maternal and child health were poor in Pakistan. Major reasons were: communication gap between community and national health system, resource crunch, and spending the available resources on tertiary care and neglecting primary health care and rural population. Being signatory to Alma Ata declaration, the Government of Pakistan took concrete steps in collaboration with World Health Organization (WHO), and launched its first nation-wide community based health programme known as Lady Health Worker’s Program in 1994 [7]. LHWs are recruited through a strict recruitment and selection criteria laid down in the basic design of the program. After her recruitment, each LHW has to undergo 15 months training after which she is supposed to serve a population of about 1000 or 150 homes by visiting 5-7 homes on daily basis [7,8]. Currently over 100,000 LHWs are working in the country covering about 60-70% of the population which is mostly rural. The government is spending on average PKRs 44,000 per LHW, on annual basis [7,9].

The Program is directly contributing to the Millennium Development Goals (MDGs) 1, 4, 5, and 6; and indirectly to MDG 7. After the devolution of health system in Pakistan in 2011, and when the provinces are strategizing for their respective health sector programmes [10], it is an opportune time to do a stock-taking of the LHW programme. Therefore, in this paper, the National Program for Family Planning and Primary Health Care is assessed using the SWOT analysis technique. SWOT is the acronym for Strengths, Weaknesses, Opportunities and Threats. This tool identifies and assesses strengths and weaknesses of the organization. It also identifies the opportunities and threats that exist in the external environment that should be utilized and avoid respectively. It is a subjective tool in which the assessor categorizes the strengths, weaknesses, opportunities and threats as per perceptions and not by objective or quantifiable measures. The analysis provides a basis to assess the likelihood of a program’s success or failure [11]. The analysis is presented under the aforementioned four headings.

## Methods

Method used for the SWOT analysis is primarily a literature review of about 22 peer reviewed papers, searched via Google Scholar and PubMed. MeSH words used were Primary Healthcare, Human Resources for Health, Management Information System, Lady health worker, Vertical program, Developing countries, Pakistan. All those research papers were excluded which used LHWs as data collectors or studied their services rendered in the communities. Papers which were developed around the programme *per se* were included. This analysis helped in making recourse to program structure, dynamics and reports documented so far.

## Review

a) **STRENGTHS:** Panel 1 gives an account of the strengths.

Panel 1: STRENGTHS

• Political commitment

• Recruitment and Selection procedures

• Wide coverage outreach – rural areas focused

• Integrations with healthcare system at upper levels

• Defined management and supervisory structures

• Comprehensive healthcare provision

• Management Information System (MIS)

• Training of LHWs part of the system

• Positive impact on health indicators

• Cost effective intervention

### Political commitment

It is heartening to see that the LHW programme received adequate political commitment, no matter which regimen was in power, military or democratic since 1994. There has been a wide recognition of the programme among the political arena and all government quarters. The financial and administrative support has continued without any interruption.

### Recruitment and selection

The main strength of this nation-wide coverage has been attributed to program strategy of rapidly recruiting, training, and deploying community based female workers primarily identified by the community itself [12]. The process enables the communities to identify appropriate females for jobs providing sense of ownership to the communities. This ensures empowerment of women selected for the job, thus improving their social status, quality of life and overall livelihood.

### Wide coverage

It is one of the largest community based programs covering up to 60-70% of the population comprising mainly the rural poor through regular outreach activities.

### Integration

The program is loosely integrated into existing health system at least at the higher tiers.

### Management and supervisory structures

The program is comprehensively designed with implementation strategies and is also blessed with well-planned management and supervisory structures ensuring regular and periodic monitoring and evaluation. Each LHW is supervised through supportive supervision by the lady health supervisors (LHS), District Coordinator, Assistant District Coordinator, Provincial Field Programme Officer and Executive District Officer (Health). However, the immediate and regular supervision is the responsibility is of LHS. An LHS is supposed to supervise 20 to 25 LHWs during the month and visits each LHW at least once a month in her health house and assess her work through records and physical verification in randomly selected households from the treatment register of the respective LHW. The LHW program demonstrates a phased scale-up with a conscious focus on program management. It has succeeded at reaching scale and is integrated into existing public health–system structures through clear policy planning. There is significant political will and some long-term financial support from the federal government for this program, which provides the potential for each layer of management to be coordinated and funded [13].

### Comprehensive health care

LHWs program provides preventive and curative healthcare at the doorstep of the population. The PHC services package include: treatment of minor ailments and referral to the first level care facilities (FLCF) when required, registration of pregnant mothers for antenatal care, ensuring clean and safe delivery, counselling of pregnant and lactating mothers in related issues, screening of neonates for problems requiring referral, weight monitoring of the children under three years of age, counselling regarding breast feeding and weaning, counselling of eligible couples regarding family planning, and provision of medicines and contraceptives to patients and clients. This makes the program effective in achieving health status goals [13].

### Management information system

The backbone of an effective and efficient management of any program is its information system. LHW program has its own comprehensive management information system (MIS) called LHW-MIS. The program’s MIS not only records data about all PHC activities and logistics, but also has a regular system of its transmission to district, provincial and federal levels. The information gathered through nine tools not only helps the LHW to keep track of health status of her catchment population, but also helps supervisors and managers of the program to assess the performance of the respective LHW. Moreover, it collects information at household level, thus accounting for clients going to both private as well as government health facilities [7].

### Training

Facility based and paid training of LHWs has been effective and efficient with recognized curricula and other protocols beside regular refresher courses.

### Impact of the program

Community health workers have made a measureable impact on health indicators by bridging the gap between community and the health care delivery system, enhancing health service utilization and creating awareness about health practices among people through health education [4]. Oxford Policy Management in its evaluation report documented significant difference in health indicators in areas covered by LHWs as compared to non-covered areas. The demographic and health survey of 2006-2007 also mentioned prominent improvement in infant mortality rate (IMR), maternal mortality ratio (MMR) and contraception prevalence rate (CPR) in areas covered by LHWs [7,8]. The fourth comprehensive review of the program found that as compared to communities not served by the LHWs, the served households were 11% more likely to use modern family planning methods, 13% were more likely to have had a tetanus toxoid vaccination, 15% more were likely to have received a medical check-up within 24 hours of a birth, and 15% more were likely to have immunized children below three years. The improvements in health indicators among the populations covered by the LHWs were not attributable to the program alone. Researchers had noted that other positive changes such as economic growth, increased provision of health services, and better education services helped to enhance the impact [11].

### Cost effective intervention

Lady health worker’s services including her salary, costs around US$750 and whereby she covers a population of 1000 people. So services delivered by a lady health workers are highly cost effective (around 75 cents per person) in a poor setting and in a resource constrained health system [14].

b) **WEAKNESSES:** The weaknesses of the program are identified in panel 2.

Panel 2: WEAKNESSES

• Poor management at lower level

• Poor integration at lower levels

• Problems in salaries payment

• Job insecurity

• Weak supplies and equipment provision

• Weak referral systems

• Poor integration of MIS with health system

• Poor supervision and linkages with peripheral health facilities

• Low quality care in some parts

• Sinecure contingents

• Slow progress in meeting targets

• Less impact in areas like sanitation and breast feeding

### Management and integration

Despite the phased rollout and well-planned management and supervisory structures, management of the LHW program faces several challenges. Frequent turnover among supervisory and logistics staff precludes the development of expertise among senior staff to guide the evolution of the program and ensure quality of care [15]. In addition, performance monitoring reports have revealed limited or uneven integration with BHUs and other health-related programs, depending upon the level of functioning of the pre-existing Women’s Health Committees and BHUs. LHW program should be coherently inserted in the wider health system [1]. In other areas, there is a significant contingent of LHWs that provide low quality of care or do not work. This may in part be due to delays in planned improvements targeted at management and organizational development [11,16].

### Salaries and jobs insecurity

Recently there have been delays in paying salaries to LHWs across the country. Widespread protests and demonstrations by LHWs have attracted media coverage, which is not good for the image of the program; and motivation of the present and prospective workforce [17]. Moreover, the salary is still called stipend not progressive like other jobs. There is job insecurity and in 18 years since its inception the LHWs have not attained the status of government employees. Though it might be against the spirit of barefoot doctors’ philosophy but is needed to keep the workforce motivated.

### Involvement in other public health interventions

LHWs are overworked due to their involvement in other public health activities launched by donor agencies or NGOs or by the health department e.g. EPI, TB DOTS, Malaria, etc [18]. Due to this overburdened job description and additional duties, her primary mandate i.e. primary health care, family planning, antenatal and postnatal care, advise on nutrition and immunization of mother and child etc. is at stake.

### Weak systems in supplies and equipment provision

There are deficiencies in the disbursement of funds and supplies at all levels [11]. In spite of its large expansion, evaluators have found that serious weaknesses in the provision of supplies, equipment and referral services need to be addressed on urgent basis [16].

### Weak referral system

In a comprehensive review of global community based health programs, the Global Health Workforce Alliance in 2010 noted that the program suffers from weak referral systems, possibly due to rapid integration into a weak national health system. Such weaknesses result from poor planning and lead to problems of sustainability, especially in both quality of care and retention of health workers [19].

### Poor integration of MIS

Though there is an MIS but it is not integrated with overall health system [14]. This leaves a vacuum in decision making because the problems and issues from the grass root level are not taken into consideration while making allocations, disbursements, procurements etc.

### Poor supervision and linkages with peripheral health facilities

Though there are good linkages at higher levels, at the field levels i.e. BHUs and other areas, the linkages are poor due to inherent weaknesses in the health system itself.

### Low quality care/sinecure contingents of LHWs

There are low quality services provided by LHWs in some parts of the country. In other parts there are contingents of LHWs drawing salaries but not working making the overall performance of LHWs program weak.

### Slow progress

The progress of the program has remained slow in achieving its targets.

### Impact

There has been less success in the areas of health knowledge, sanitation, and key behaviours such as exclusive breastfeeding for the first 6 months of life [3,13]. Less impact is also visible in areas such as enhancing health knowledge, sanitation, exclusive breast feeding, and neonatal mortality.

c) **OPPORTUNITIES:** Panel 3 identifies some opportunities for the program.

Panel 3: OPPORTUNITIES

• Wide coverage and social acceptability

• Training capacity can be used by others

• Emergency obstetrical care training for some LHWs

• Health system research

• Use for women empowerment

• Use for poverty alleviation strategies

### Wide coverage

The large coverage of the program and robust workforce can afford opportunities for future public health interventions. Given the fact that women in remote rural areas are in need of permission to seek health care from female providers only, LHWs can be instrumental in transforming the health care seeking practices and behaviours [20].

### Training in emergency obstetrics care

Some LHWs based on their performances could be trained and certified in providing obstetrical care of basic type. Community health workers have been used in many settings for plugging the gaps in service delivery, when skilled personnel cannot be deployed for any reason [21].

### Health system research

The LHWs program wider reach and numbers of LHWs available can be made part of a workforce that could be regularly used as team for health system research.

### Women empowerment

The program can be used as a springboard for the community women empowerment. LHWs can very well organize the community by developing women groups and Health committees in their area -an important aspect of the primary health care approach [22]. LHWs themselves have emerged as community leaders in a context where women are given minimal space in the local politics.

### Strengthening referral system

One of the weaknesses of the primary health care program has always been its poor and weak referral system. LHWs can be instrumental in strengthening the referral of vulnerable patients particularly the women and children to ensure timely and appropriate health care seeking to save lives [23].

d) **THREATS:** Threats are depicted in panel 4.

Panel 4: THREATS

• Poverty, patriarchy and social norms

• Political interference

• Lack of funds

• Political and social environment

• Non-acceptance by established medical professions

• Quackery implications

### Poverty, patriarchy and social norms

LHW has struggled and will be facing the daunting challenge of prevailing poverty which is the major constraint in promoting healthy behaviours among the poorest communities they serve. Moreover, patriarchal structure of society compounded with variety of social norms impedes her own social mobility and jeopardizes her place in the milieu [24].

### Political interference

Due to huge opportunities of employment, the program is vulnerable to political interference.

### Funds

After the 2011 devolution, the financing mechanism of the programme has still to be decided at the provincial level. Till then, lack of financial support by the government may be dangerous for the health of the program in the long run. It is though hoped that the provinces will soon strategize for the fate of the programme funding, there is a visible unrest amongst the workforce, which is affecting the day to day services to the poor and remote communities.

### Political and social environment

In some parts of the country there is non-acceptance of female gender to hold sway. Recent killings of some LHWs in anti-polio campaigns are alluding to ominous future [25]. Even though the program’s focus is maternal and child health care, yet LHWs have to face difficulties owing to traditional gender norms in many parts of the country [26].

### Non-acceptance by established professions

The established professions of doctors, nurses, lady health visitors may act as obscurantist forces in the progress of the program.

### Quackery

Lady Health Workers may fall prey to the temptation of inappropriate practices in the private sector that may erode public confidence in the program.

## Discussion

The government has so far shown a great deal of political commitment for the continuity of the program. It has been proved to be a cost effective venture to make the primary health care at the doorstep of the poor, remote and rural population. The program by virtue of its comprehensive design, planning, implementation and supervision mechanisms aided by an MIS, can deliver results even better now in the post devolution scenario, whereby provinces now have a direct control over the personnel of the program. This program has the potential to improve the health indicators and creating an impact at the primary health care level in the health system of Pakistan [27]. However, a referral system will have to be formalized and incentivized. Nonetheless, such an extensive community based program has a definite potential to improve the mother and child survival as has been demonstrated in other parts of the world, yet the weaknesses in the program ought to be addressed to take the best productivity out of the community based health workers [28]. The provinces must work on widening the coverage of services, and invest on its potential contribution to health system research. To a certain extent, the program has shown an impact on women empowerment and poverty alleviation [24,29]. An early integration of the program on provincial health strategies and a clear road map for the workers’ career will not only avoid the unnecessary politicization of the program but will enhance the level of confidence and motivation of the lady health workers [30]. Two way linkages have to be strengthened more: with the communities and to first level and secondary level care facilities for timely referrals of the complicate cases [5]. Closer supervision and performance management will also improve the deliverables of the workers, geared towards improving the mother and child health indicators in the country [16,23].

## Conclusion

In order to make efficient and effective use of the most robust primary healthcare workforce in Pakistan, i.e. the lady health workers, more political commitment is needed at the highest levels. The program needs to be integrated properly into the existing health system along with predictable and just remuneration structures for the lady health workers. Mechanisms need to be developed at the earliest to provide job security to the workers and to increase their motivation.

## Abbreviations

BHU: Basic health unit; CHW: Community health worker; CPR: Contraceptive prevalence rate; EPI: Expanded program of immunization; FLCF: First level care facility; ICPD: International conference on population & development; IMR: Infant mortality rate; LHS: Lady health supervisor; LHW: Lady health worker; MDG: Millennium development goals; MIS: Management information system; MMR: Maternal mortality ratio; NGO: Non-governmental organization; PHC: Primary health care; SWOT: Strengths weaknesses, opportunities, threats; TB-DOTS: Tuberculosis-directly observed treatment short course; WHO: World health organization.

## Competing interests

Authors do not have any competing interests to declare.

## Authors’ contributions

“MSW and BTS conceptualized the theme of the paper; MSW carried out the literature review, MSW and BTS synthesized the literature; AA participated in the write up and drafting of the manuscript; BTS added to the intellectual content of the paper. All authors read and approved the final draft of the manuscript.”

## References

[B1] Bhutta ZA Lassi ZS Pariyo G Huicho L Global experience of community health workers for delivery of health related millennium development goals: A systematic review, country case studies, and recommendations for integration into national health systems 2010 Geneva: Global Health Workforce Alliance, World Health Organization

[B2] Khan MH Saba N Anwar S Baseer N Syed S Assessment of knowledge, attitude and skills of lady health workers Gomal J Med Sci 2006 4 2 57 60

[B3] Liu A Sullivan S Khan M Sachs S Singh P Community health workers in global health: scale and scalability Mount Sinai J Med 2011 78 419 435 10.1002/msj.20260 21598268

[B4] Haq Z Hafeez A Knowledge and communication needs assessment of community health workers in developing countries: a qualitative study Hum Res Health 2009 7 59 10.1186/1478-4491-7-59 PMC2720909 19622172

[B5] Afsar HA Qureshi AF Younus M Gulb A Mahmood A Factors affecting unsuccessful referral by the lady health workers in Karachi, Pakistan J Pak Med Assoc 2003 53 521 528 14738257

[B6] World Health Organization: Declaration of Alma-Ata International Conference on Primary Health Care 1978 USSR: Alma-Ata

[B7] Hafeez A Mohamud BK Sheikh MR Shah SAI Jooma R Lady health workers programme in Pakistan: challenges, achievements and the way forward J Pak Med Assoc 2011 61 210 215 21465929

[B8] Mahmood A Naz SS Assessment of management information system (MIS) of National Program for Family Planning and Primary Health Care [LHW Program] 2012 Islamabad: Population Council

[B9] Jalal S The lady health worker program in Pakistan-a commentary Eur J Public Health 2011 1 2 10.1093/eurpub/ckq199 21278131

[B10] Government of Pakistan The 18th Amendment to the Constitution of the Islamic Republic of Pakistan 2011 Islamabad http://pakistanconstitutionlaw.com/18th-amendment-2010/ (accessed on November 10th, 2013)

[B11] Zuckerman AM Healthcare strategic planning 2012 3 Chicago: Health Administration Press

[B12] Bhutta ZA Pariyo G Huicho L Global Experience of Community Health Workers for Delivery of Health -Related Millennium Development Goals: A Systematic Review, Country Case Studies, and Recommendations for Integration into National Health Systems 2010 Geneva: World Health Organization

[B13] Afsar HA Younus M Gul A Outcome of patient referral made by the lady health workers in Karachi, Pakistan J Pak Med Assoc 2005 55 209 211 15960288

[B14] World Health Organization Country case study: Pakistan’s lady health worker programe GHWA Task Force on scaling up education and training for health workers 2008 Geneva: Global Health Workforce Alliance

[B15] Shaikh BT Reza S Afzal M Rabbani F Gender sensitization among health providers and communities through transformative learning tools: experiences from Karachi, Pakistan Edu Health 2007 7 118 18080960

[B16] Oxford Policy Management Lady Health Worker Programme: Third party evaluation of performance 2009 Oxford, UK: Oxford Policy Management

[B17] Farooq A Lady health workers rally baton charged tear-gased 2012 Lahore: Daily Times

[B18] Haq Z Iqbal Z Rahman A Job stress among community health workers: a multi-method study from Pakistan Int J Ment Health Syst 2008 2 15 10.1186/1752-4458-2-15 18954470 PMC2584063

[B19] Bhattacharyya K Winch P LeBan K Tien M Community Health Worker Incentives and Disincentives: How They Affect Motivation, Retention, and Sustainability 2001 Arlington, VA: US Agency for International Development, Basic Support for Institutionalizing Child Survival Project (BASICS II)

[B20] Shaikh BT Haran D Hatcher J Women’s social position and health seeking behaviors: is healthcare system accessible and responsive in Pakistan? Healthcare Women Int 2008 29 8/9 945 959 10.1080/07399330802380506 18726800

[B21] Standing H Chowdhury HM Producing effective knowledge agents in a pluralistic environment: what future for community health workers? Soc Sci Med 2008 66 10 2096 2107 10.1016/j.socscimed.2008.01.046 18342421

[B22] World Health Organization Pakistan’s experience in Lady Health Workers (LHWs) Programme. Regional Committee for the Eastern Mediterranean. 51st session 2004 Cairo: Agenda item 6(c) EM/RC51/12

[B23] Abrejo FG Shaikh BT Saleem S ICPD to MDGs: the missing links and the common grounds Reprod Health 2008 5 4 10.1186/1742-4755-5-4 18783600 PMC2546384

[B24] Khan A Women’s empowerment and the lady health worker programme in Pakistan 2008 Karachi: Collective for Social Science Research

[B25] Two polio vaccination workers have been killed in north-western Pakistan in the latest of a spate of deadly attacks[accessed on March 19, 2013] Available from: http://www.bbc.co.uk/news/world-asia-20779388

[B26] Mumtaz Z Salway S Waseem M Umer N Gender-based barriers to primary health care provision in Pakistan: the experience of female providers Health Policy Plan 2003 18 261 269 10.1093/heapol/czg032 12917267

[B27] Shaikh BT Do we need to be skeptical about millennium development goals? Pak J Public Health 2011 1 1 56 58

[B28] Haines A Achieving child survival goals: potential contribution of community health workers Lancet 2007 369 2121 2131 10.1016/S0140-6736(07)60325-0 17586307

[B29] Closser S Jooma R Why we must provide better support for Pakistan’s female frontline health workers PLoS Med 2013 10 10 e1001528 10.1371/journal.pmed.1001528 24115914 PMC3792984

[B30] Mazhar A Shaikh BT Reforms in Pakistan: decisive times for improving maternal and child health Healthcare Policy 2012 8 1 24 32 23968601 PMC3430152

